# Recent advances in understanding the role of the basal ganglia

**DOI:** 10.12688/f1000research.16524.1

**Published:** 2019-01-30

**Authors:** Kristina Simonyan

**Affiliations:** 1 Department of Otolaryngology, Massachusetts Eye and Ear Infirmary, Boston, MA, USA; 2 Department of Neurology, Massachusetts General Hospital, Boston, MA, USA; 3 Harvard Medical School, Boston, MA, USA

**Keywords:** basal ganglia, somatotopy, extrinsic network, intrinsic network

## Abstract

The basal ganglia are a complex subcortical structure that is principally involved in the selection and implementation of purposeful actions in response to external and internal cues. The basal ganglia set the pattern for facilitation of voluntary movements and simultaneous inhibition of competing or interfering movements. In addition, the basal ganglia are involved in the control of a wide variety of non-motor behaviors, spanning emotions, language, decision making, procedural learning, and working memory. This review presents a comparative overview of classic and contemporary models of basal ganglia organization and functional importance, including their increased integration with cortical and cerebellar structures.

## Introduction

The basal ganglia are a group of interconnected subcortical nuclei that include the putamen and caudate nucleus (collectively, the striatum), globus pallidus (its internal GPi and external GPe segments), substantia nigra (its pars compacta SNc and pars reticulata SNr), and the subthalamic nucleus (STN). The limbic portion of the basal ganglia is composed of the nucleus accumbens, ventral pallidum, and ventral tegmental area. The basal ganglia are principally involved in the selection and implementation of purposeful actions in response to external and internal cues. Most prominently, the basal ganglia set the pattern for facilitation of voluntary movements and simultaneous inhibition of competing or interfering movements ^1, 2^. Their contribution is also linked to the control of a wide range of complex non-motor behaviors, including emotions, language, decision making, procedural learning, and working memory.

Contemporary views about the expanded structural and functional organization of the basal ganglia are informed by several key discoveries that have been made in the past few years. These have important implications not only in regard to normal functioning of the basal ganglia and larger neural networks in general but also in terms of unraveling piece-by-piece yet-unknown mechanisms of various neurological and psychiatric disorders, such as Parkinson’s disease, dystonia, obsessive–compulsive disorder, and Tourette syndrome, to name a few. This review presents a comparative overview of classic and contemporary models of basal ganglia organization, including their increased envelopment with cortical and cerebellar structures, and a discussion of the functional importance of basal ganglia and their significance in brain disorders.

## Intrinsic basal ganglia connectivity

**Figure 1.**
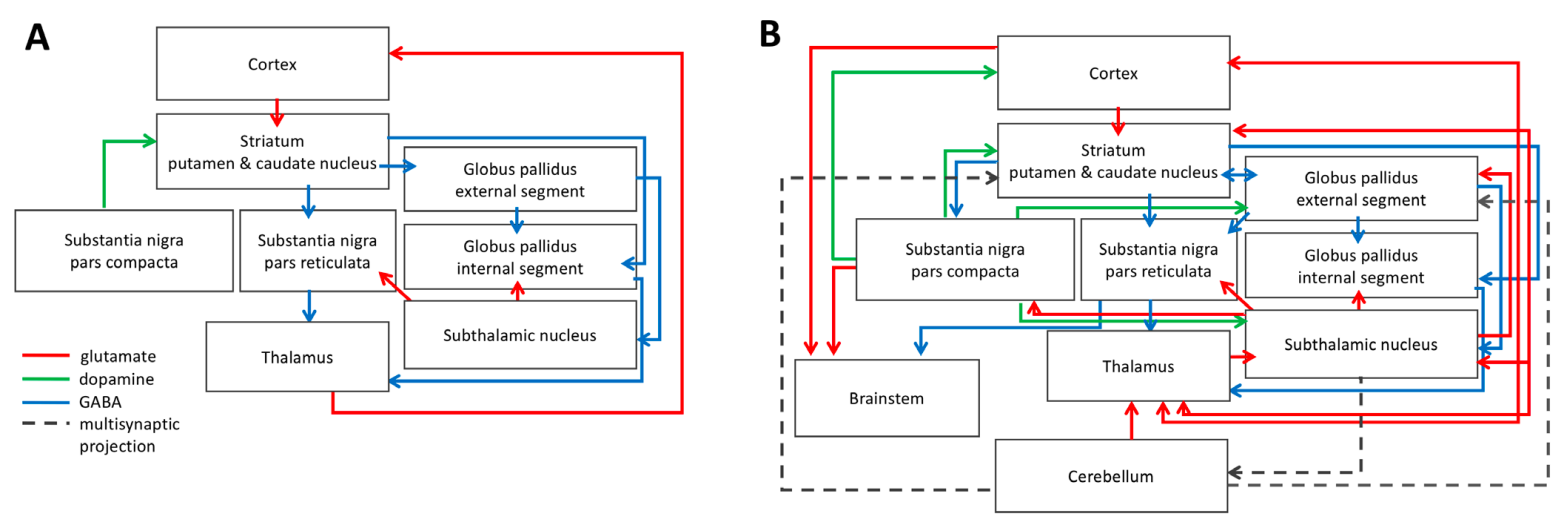
Schematic representation of basal ganglia intrinsic and extrinsic connectivity according to ( **A**) the classical model and ( **B**) the contemporary model. Modified and adapted with permission from Simonyan *et al*. ^7^.

There are several levels of complexity in the organization of basal ganglia. Since the late 1980s, the classic model of the basal ganglia in both humans and animals has been built on the presence of intrinsic direct and indirect pathways, both comprising a consecutive set of excitatory glutamatergic and inhibitory GABAergic projections. The intrinsic model ^3^ includes top-down cortical projections to the striatum, which further converge on GPi and SNr either directly or indirectly via GPe and STN ( Figure 1A). The output from GPi and SNr is then directed to the thalamus, which further projects back to the cortex, forming a complete cortico-basal ganglia-thalamo-cortical loop. Both direct and indirect basal ganglia pathways are modulated by endogenous dopamine release from the SNc, which acts upon dopamine D _1_-family receptors expressed on medium spiny neurons (MSNs) predominantly within the net excitatory direct pathway and D _2_-family receptors expressed predominantly within the net inhibitory indirect pathway, thus balancing excitation and inhibition within the thalamo-cortical circuitry.

Building on the framework of this original model, recent studies discovered a much denser intrinsic basal ganglia connectivity ( Figure 1B). One of the important updates to this model was the identification of bridging collaterals between the direct and indirect pathways, providing evidence for a cross-talk between these circuits, which were initially assumed to be largely segregated ^4– 6^. The density of bridging collaterals was found to modulate the functional balance within the basal ganglia such that an increase in bridging collaterals led to enhanced pallidal inhibition ^6^. In line with these findings, a recent positron emission tomography (PET) study in human subjects demonstrated a great degree of overlap between the direct and indirect basal ganglia pathways ^7^.

Another revision to the classical model of basal ganglia involved the expansion of STN’s responsibility within its network. The STN is now considered a major input relay station receiving direct projections from various cortical and subcortical regions, including the recently identified hyperdirect cortico-subthalamo-pallidal pathway ^8– 10^. The paramount significance of this finding is in its clinical relevance to the treatment of brain disorders, such as Parkinson’s disease, where STN is a target for deep brain stimulation (DBS). Thus, detailed knowledge of its involvement in extrinsic cortical (hyperdirect pathway) and intrinsic basal ganglia (direct and indirect pathways) networks provides advanced knowledge, allowing fine-tuning of the DBS procedure and programming.

The reciprocity between basal ganglia structures is further established by expanded circuitry of GPe, which not only projects downstream to STN but also sends direct collaterals to GPi and SNr as well as feedback projections to the striatum ^11, 12^. GPe has been found to contain heterogeneous populations of neurons, including Arky-GPe neurons targeting striatal GABAergic interneurons ^13^, Lhx6-GPe neurons strongly projecting to the SNc and STN, and PV-GPe neurons predominantly projecting to the STN and parafascicular thalamic nucleus ^14^, which collectively contribute to distinct motor and non-motor behaviors via different pallidal circuits ^15– 17^. Overall, this higher level of integrity and interactions between basal ganglia structures allows their enhanced functional importance in contributing to and controlling an array of human and animal behaviors.

Although the SNc is known as the main structure harboring dopaminergic neurons that project to different basal ganglia and cortical divisions, dopaminergic neurons were also found to be scattered throughout the primate striatum and abounded in its ventral portion ^18– 20^. An increase in the number of striatal dopaminergic neurons has been observed as a potentially compensatory response to the loss of nigrostriatal dopaminergic innervation, with the significance of implications for neurological disorders involving the basal ganglia and abnormal dopaminergic function, such as in Parkinson’s disease and dystonia. However, a recent study has challenged these assumptions by defining the vast majority of striatal tyrosine hydroxylase (TH) interneurons in transgenic enhanced green fluorescent protein (EGFP)-TH mice as medium-sized, aspiny, or very sparsely spiny interneurons expressing low levels of TH and making GABAergic synapses onto spiny projection neurons ^21^.

**Figure 2.**
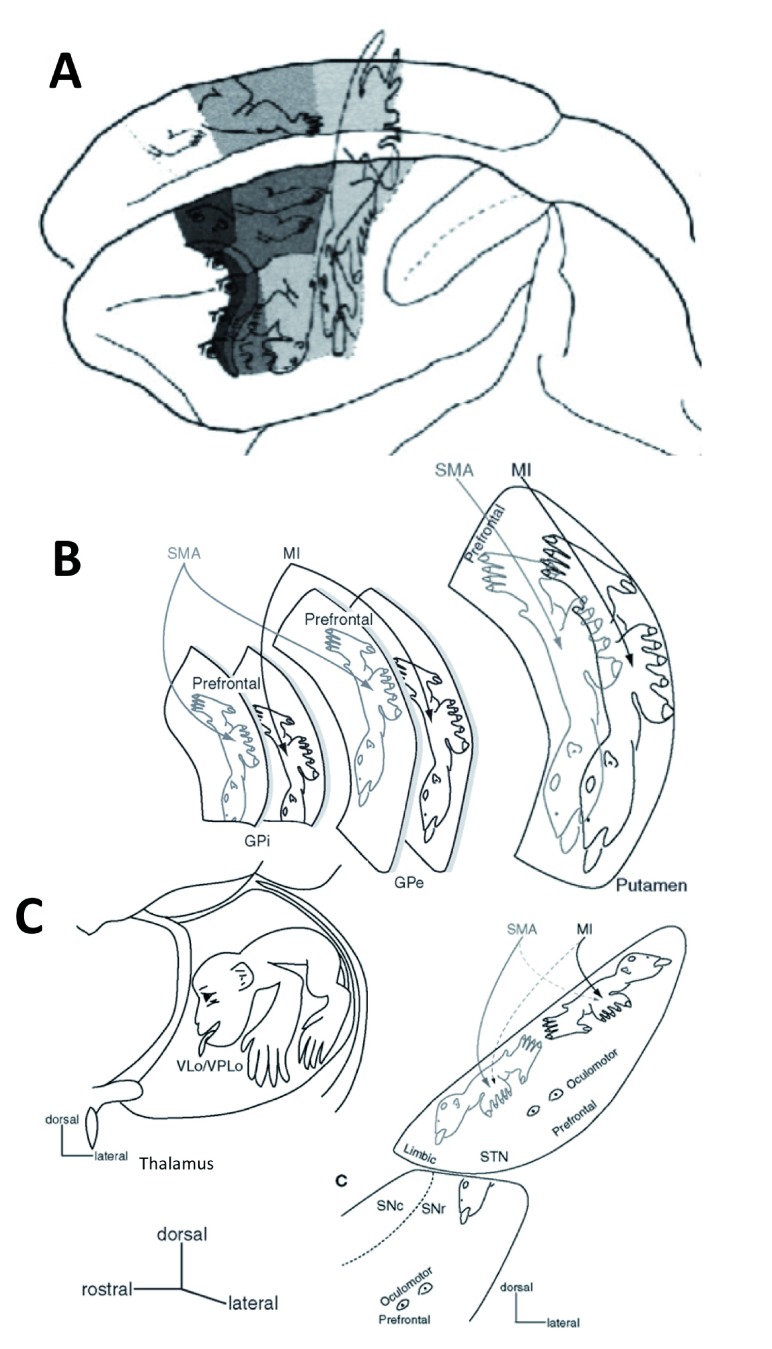
Somatotopical representations within the motor cortex, basal ganglia, and thalamus ( **A**) Lateral and medial view of the monkey brain showing the somatotopic representation of body regions. Light-gray shading indicates primary motor cortex, and dark-gray shading indicates premotor cortex. Adapted with permission from Fadiga *et al*. ^49^. ( **B, C**) Dorsoventral views of the basal ganglia subdivisions ( **B**) (putamen, external segment of the globus pallidus [GPe], internal segment of the globus pallidus [GPi], substantia nigra pars reticulata [SNr], and substantia nigra pars compacta [SNc]) and thalamus ( **C**) depicting somatotopic body representations. Adapted with permission from Nambu ^28^.

In addition to these discoveries of cellular composition of the basal ganglia, an important feature of intrinsic organization is that their input is arranged in a highly topographic manner ( Figure 2). Similar to the distribution of body regions within the sensorimotor cortex, the basal ganglia nuclei are too somatotopically organized, harboring leg-hand/arm-face-larynx representations ^22– 28^. These areas receive projections from corresponding motocortical regions, with the somatotopy preserved at the entire rostro-caudal extent and in their output to the thalamus, which loops back to the corresponding cortical representations. In the striatum, pallidum, and thalamus, the distribution of body regions is along the dorsal–ventral axis, with primary motor and premotor cortical projections forming parallel homunculi. The STN, on the other hand, forms a mirror set of homunculi, with the primary motor cortex predominantly projecting to its lateral part and the premotor cortex predominantly projecting to the medial part, although some areas receive a convergent input from both motocortical regions. Finally, SNr and SNc projections with the striatum have an inversed dorsoventral topography, such as the dorsal parts of the striatum project to the ventral SN regions, while the ventral striatal regions project dorsally. The dorsal one-third of SN carries the orofacial representation as a continuation of the same regions of the GPi, and the more ventral region receives an input from the premotor territories of the putamen ^29, 30^. The rostromedial two-thirds contain projections from prefrontal striatal areas ^31^, and the most medial part receives limbic striatal input ^32^. The SNc dopaminergic neurons give rise to topographically organized striatal projections ^33– 36^. Specifically, the dorsal tier and the most medial part of the ventral tier of the SNc project to the ventromedial striatum; the remaining ventral tier projections are directed to the associative striatum, and the ventrally extending cell columns of the ventral tier are connected to the sensorimotor striatum ^37, 38^. The nigrostriatal connections, at least those of the striatal matrix, are reciprocal, and dopaminergic neurons synapse on MSNs, which in turn reach back to the somata and dendrites of SNc neurons ^39, 40^. However, reciprocal nigrostriatal connections do not form a closed loop; instead, the ventral tier of SNc that receives input from the ventromedial striatum projects to the more dorsolateral striatum ^41, 42^. In addition, while the nigral projections are topographically organized and directed to a particular striatal region, the extending weaker fibers also reach all other striatal divisions ^43^. Thus, such feedforward connections allow the interplay between different striatal divisions that are responsible for the control of different aspects of a behavior.

## Extrinsic connectivity of the basal ganglia

**Figure 3.**
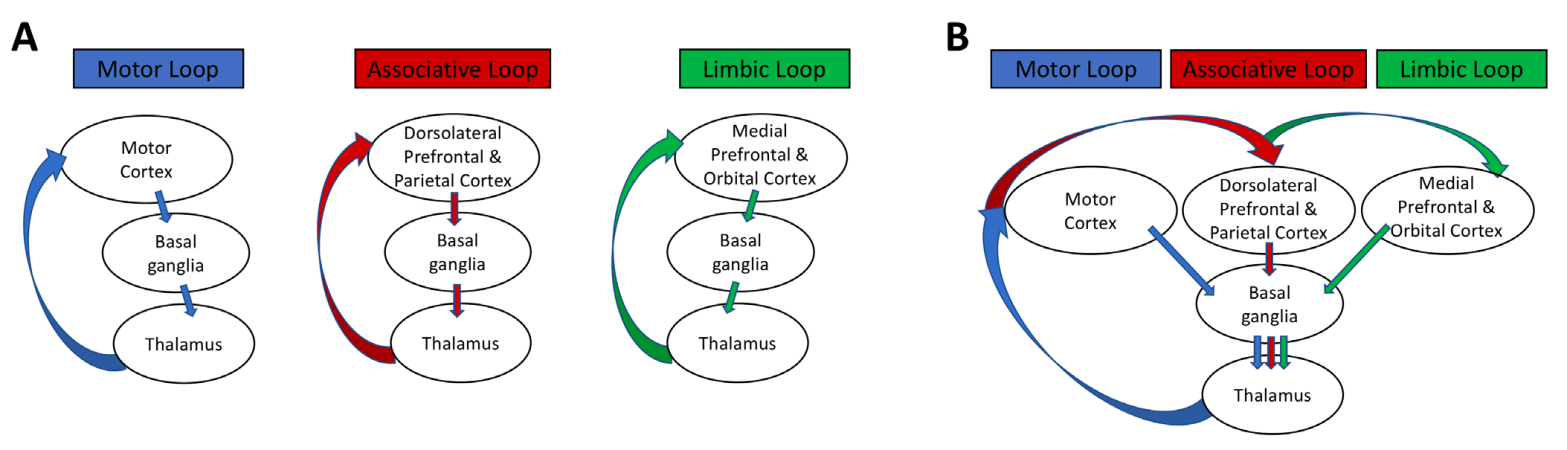
Schematic representation of major basal ganglia loops: the motor, associative, and limbic. The representation is organized according to ( **A**) the parallel-projecting hypothesis ^55– 58^ and ( **B**) information convergence across the loops. Adapted from Percheron and Filion ^59^.

Originally, there were two different proposals of how information may flow within the extrinsic cortico-basal ganglia-thalamo-cortical pathways. The prevailing view included the formation of parallel-projecting loops ^8^, whereas the alternative view pertained to information convergence across the loops ^9^ ( Figure 3). The three principal functional loops are the motor loop, which projects via motor and premotor cortices; the associative loop, which involves dorsolateral prefrontal and parietal cortices; and the limbic loop, which converges on orbital and medial prefrontal cortex. However, these functional loops are found to be only partially segregated while establishing the anatomical links at different cortical, striatal, pallidal, and subthalamic levels ^44^. In addition, selection and processing of a complex goal-directed behavior require an integration across different loops that carry information about motor, cognitive, and limbic components. As such, both parallel processing and information convergence are present within the cortico-basal ganglia-thalamo-cortical pathways.

In regard to extrinsic basal ganglia neuromodulatory projections, it has recently been demonstrated that, in addition to the major excitatory (glutamatergic) cortico-basal ganglia input ^45^, the striatum receives direct GABAergic projections from motor and auditory cortices ^46^. Primary and secondary motor cortex-striatal long-range projections in mice are supported by different molecular subtypes of GABAergic neurons, which express either somatostatin (SOM ^+^) or parvalbumin (PV ^+^) and differ in their target cell preference and the modulatory effects of a motor behavior ^47^. On the other hand, while it is established that the basal ganglia modulate the cortex indirectly via the inhibitory (GABAergic) output to thalamus, a direct GABAergic/cholinergic projection between the GPe and frontal cortex does also exist ^48^. Again, two cell types comprise this connectivity and differ in their electrophysiological properties, cortical target projections, and expression of choline acetyltransferase (ChAT).

Another drastic revision to the organization of extrinsic basal ganglia connectivity is the addition of the cerebellum to this circuitry. The original view of the cerebellum and basal ganglia was that of a “funneling” system, where both structures receive and process information from prefrontal, parietal, and temporal areas with a subsequently integrated output to the primary motor cortex for execution of a motor command ^50– 54^. However, the development of a retrograde transneuronal tracer, herpes simplex virus type 1 (HSV1), allowed the important discovery that different subdivisions of the basal ganglia (that is, GPi and SNr) and cerebellum (that is, dentate nucleus) have, in fact, a widespread output via distinct target thalamic nuclei to other cortical regions, such as subdivisions of premotor, oculomotor, prefrontal, and inferotemporal areas. Based on a revision of anatomical connectivity, it was proposed that the basal ganglia and cerebellum influence not only the motor behaviors but also various cognitive and limbic functions ^60– 67^. However, it was still believed that the basal ganglia-thalamo-cortical and cerebello-thalamo-cortical pathways form anatomically independent loops that converge and communicate mainly at the level of target cortical regions ^68, 69^.

Further modifications to this view came with the development and use of another retrograde transneuronal tracer, the rabies virus, which led to the discovery of basal ganglia and cerebellar connectivity at the subcortical level ^70– 75^. Specifically, the STN was identified as an output region of dense disynaptic projections via the pontine nuclei to the cerebellar cortex. On the other hand, the dentate nucleus was found to be the main output structure, primarily via intralaminar thalamic nuclei, of dense disynaptic projections to the striatum and trisynaptic projections to the GPe but not the GPi. In addition, other deep cerebellar nuclei (that is, fastigial and interpositus nuclei) were demonstrated to be sources of striatal disynaptic projections, albeit to a lesser extent than the dentate nucleus. Given the participation of the GPi and GPe in the intrinsic direct and indirect basal ganglia pathways, respectively, it was suggested that the cerebellar output may preferentially influence the indirect basal ganglia pathway ^71, 76^. Similar to the basal ganglia-thalamo-cortical output, the basal ganglia-cerebellar anatomical network is topographically organized in such a manner that the motor, associative, and limbic regions are interconnected between the two structures. This bidirectional communication between the basal ganglia and cerebellum at both subcortical and cortical levels is assumed to provide a backbone of the integrated functional network where motor and non-motor information is processed at multiple stages before its final cortical output.

In conclusion, the development of new methodologies and the conduct of parallel research in animal models and human subjects paved the way for a more complex view of basal ganglia structural organization. These advances have enhanced our understanding of the functional importance of this structure within the large-scale brain network, expanding their role in both motor and non-motor domains. Importantly, detailed knowledge of basal ganglia organization informed our views of their contribution to the pathophysiology of a range of neurological and psychiatric disorders and played a critical role in the development of novel therapeutic opportunities targeting specific anatomical or functional links (or both) of basal ganglia connectivity. Continued progress in the field of basal ganglia research will further refine and characterize the multi-layer organization of this structure, including both intrinsic and extrinsic connectivity.
